# Book Reviews

**Published:** 1904-03

**Authors:** 


### BOOK REVIEWS.

A Textbook of Practical Gynecology for Practitioners and Students. By D. Tod Gilliam, M. D., Professor of Gynecology in Starling Medical College. Columbus; Gynecologist to St. Anthony and St. Francis Hospitals, Columbus, O. Octavo, pages 650. Illustrated with 350 engravings, a colored frontispiece and full page half-tone plates. Philadelphia: F. A. Davis Company. 1903. (Cloth, $4.00 net; half-Russia, $5.00 net, delivered.)

The year 1903 will take rank as the banner year for the publication of new works on genecology; at least not in many years, if ever, have so many treatises on this branch of medicine issued from the press in a single year as during the one just ended. It is a great satisfaction to observe that they have all been of excellent character, and most will take permanent places in the literature of medicine. The author of the book before us has long been known as one of the foremost teachers and writers in this country, and it seems appropriate that he should put forth a complete treatise on the branch that he has made the subject of special study and successful practice for many years. His work, therefore, may be regarded as the result of mature experience, and it records demonstrated fact, rather than theory,—clinical results, not debatable propositions.

Gilliam divides his book into fifty chapters of nearly uniform length, his object being to make it correspond to the usual number of teaching hours devoted to gynecology in the average collegiate term. He begins with a brief chapter on the causes of disease in women, and then takes up gynecologic examination, which forms the subject material of two chapters. His directions are excellent, though in one or two respects he is, perhaps, unnecessarily minute,—a good fault, however, if it be a fault. His technic is beyond criticism. The chapters on diseases of the vulva and vagina are both interesting and instructive. He treats injuries to the pelvic floor and their repair with intelligence and skill, so also genital fistulæ and displacements of the uterus.

Gilliam describes in detail (p. 209) an operation which he, himself, devised for repositing the retrodisplaced uterus and which he terms round ligament ventrosuspension. He first described the operation at the meeting of the American Association of Obstetricians and Gynecologists at Louisville in 1900. since which time it has become known as the "Gilliam Operation." Dr. Edward J. Ill, of Newark, N. J., read a paper at the last meeting of the association aforementioned, in which he so styled it both in the text and title of his contribution. The various steps of the operation are clearly and artistically illustrated in this treatise.

The author next treats upon inflammation and trophic disorders of the cervix uteri in a well-written chapter, and then describes laceration of the cervix with the operations for its cure. Cancer of the uterus—cervix and corpus—take up several chapters, in which the most modern doctrines and procedures are taught. Fibroid tumors are considered in two chapters that are not excelled for their value on this topic, the illustrations of the operative technic in its several steps being superb. Infections of the fallopian tubes occupy attention in the next four chapters, including operation and electrical treatment of salpingitis and tubercular disease of the tubes.

Two chapters are devoted to ectopic gestation, in which the usual surgical measures are advocated. The author has clearly illustrated the several varieties of ruptures of the tube, an important item, and described a tubal abortion for which he, himself, operated. Inflammatory diseases of the ovaries, together with cystic and solid tumors thereof are dealt with in the next few chapters, which clearly describe the conditions, and in which the topic is excellently illustrated. The remaining chapters are devoted to the consideration of diseases of the urinary tract and rectum. In no treatise on diseases of women with which we are familiar have these latter topics been dealt with as elaborately and satisfactorily.

The plan of this treatise is somewhat novel and it will appeal to students and undergraduates because it is a plain, connected clinical narrative rather than a discussion of ultra scientific theories. The author's style is commendable for its simplicity and clearness. His sentences are examples of terse English; they are neither obscure nor involved, but aim at something of importance,—and hit it. The mechanical execution of the book is above criticism, and its illustrations are especially to be commended. It is a work that will take a permanent place in the libraries of those interested in the teaching or practice of gynecology.

A TEXTBOOK OF OBSTETRICS. By BARTON COOKE HIRST, M. D., Professor of Obstetrics in the University of Pennsylvania. Octavo, 900 pages, with 746 illustrations, 39 of them in colors. Philadelphia, New York, London: W. B. Saunders & Company. 1903. (Cloth, $5.00 net; sheep or half morocco, $6.00 net.)

At the present time there are several textbooks recognised as authority on obstetrics, one of which is Hirst's,—a work that stands in the van. The author makes an excellent point in his preface, wherein he declares that nearly all the diseases of women result from childbirth either simple or complicated, hence their preventive treatment rests with the obstetrician. It naturally follows, and this is also well formulated by the author, that the physician in general practice must be equally well informed in obstetrics and gynecology, at least to the extent that he may recognise the necessity for the application of this or that measure, even though he may not possess sufficient skill to make appropriate use of all; but, that the specialist in obstetrics must be an expert in the surgical treatment of diseases of women, admits of no doubt.

From the foregoing and as a corollary thereto it may be stated that the student must approach his studies through the avenue of the best authority, in the companionship of the best books. This one may be chosen with confidence and studied with the assurance that it is of the best. The changes in this edition are such as to bring it forward to the immediate present in its teachings; great attention has been paid to diseases of the genital organs following childbirth; many new illustrations have been inserted, even the kliseometer, a new instrument for determining the inclination of the pelvis, has been described and illustrated. The entire treatise commends itself to obstetrician, general practitioner, and student.

---

Transactions of the National Association of United States Pension Examining Surgeons. Second annual meeting held at Washington, D. C., May 13 and 14, 1903, including an account of first meeting held at Saratoga Springs, June 9, 1902. Edited by Wheelock Rider, M. D., Secretary. Published by the Association.

This association, which now for the first time publishes its transactions, sprang into being at Saratoga in June, 1902. It was suggested by Dr. William A. Howe of Phelps, N. Y., in a circular letter sent out to pension examining surgeons some time before, stating that it seemed feasible to organise such a body. The suggestion met with immediate favor and the result has been most satisfactory. Two meetings have been held and the work done at both is recorded in this book.

The address of welcome by the Commissioner of Pensions, Hon. E. F. Ware, delivered at the Washington meeting, May 14, 1903, is in admirable form,—witty, brief and dignified. The medical Referee, Dr. Sam Houston, also delivered an address, in the course of which he advocated the utilising of the pathologic history of instructive cases for the benefit of medical science.

Dr. Raub's paper, delivered at Saratoga, is also published in this book, and is one full of suggestive thought to pension examiners. It has been widely circulated and is eagerly sought by those who have not read it. Dr. John C. Hemmeter of Baltimore read a paper on Chronic malaria and its complications and sequelæ that is the most scientific paper in the book. It should be studied carefully by all pension examiners.

The book is well arranged and its general appearance shows the careful supervision of the secretary-editor.

---

Modern Surgery: General and Operative. By John Chalmers DaCosta, M. D., Professor of the Principles of Surgery and of Clinical Surgery in the Jefferson Medical College, Philadelphia. Octavo, 1099 pages, with over 700 illustrations, some in colors. Fourth edition, greatly enlarged and entirely reset. Philadelphia, New York, London: W. B. Saunders & Company. 1903. (Cloth, $5.00 net; sheep or half morocco, $6.00 net.)

From 1894, when the first edition of this book appeared, to 1903, the date of the fourth edition, embraces a period in which a long stride was taken by surgery. The improvements made in this work in the nine years referred to,—at first a modest manual, now a pretentious treatise,—testify abundantly to the truthfulness of this statement. If, however, we limit the time of observation to the period embraced between the editions of 1900 and 1903, we still find ample reason, through the marked progress in surgery during that time, for an entire rewriting of the fourth edition.

Besides the careful revision of the book much new matter has been added, the illustrations have been increased in number and improved in quality, while a larger sized page has been adopted, making it now one of the comprehensive textbooks on surgery, yet not cumbersome nor loaded with useless material. It deals with the present approved methods of surgical practice, and not with obsolete or antiquated details. It is emphatically a book of today and will afford satisfaction to the reader, whether teacher, practitioner, or student.

---

A Textbook on the Practice of Medicine. Designed for the Use of Students. By James Magoffin French, M. D., Lecturer on the Theory and Practice of Medicine, Medical College of Ohio; Attending Physician, St. Mary's Hospital, Cincinnati. Octavo, pp. 799. Illustrated by ten full-page plates and fifty wood engravings. New York: William Wood & Company. 1903. (Cloth, $4.00.)

If an additional treatise on the practice of medicine were needed we know of no one better qualified to supply it than Professor French. With the wealth of literature on this topic already in the hands of the profession it seems almost needless to offer another textbook at this time; nevertheless there is much reason for this one; it is compact, being of less dimensions than most of those issued recently,—an important factor for students of medicine; its make-up and general form are excellent; it is terse even to conciseness without being elementary; it deals with only the practical, leaving the theoretical behind; it contains the most modern thought and records the most recent discoveries; it omits case reports and instead deals with more useful material, thus economising space and preserving the patience of the reader; finally it is illustrated with a few well selected and excellent executed plates and engravings that give force and impact to the text. For these and other reasons, not necessary here to enumerate, we are of the opinion that this work will take an important place in medical literature.

The author writes from an experience of twenty-five years as a teacher, yet he does not intrude his own opinions dogmatically, his aim being to present the consensus of the best experience on all questions of medical practice. To this end he has drawn upon authors in textbook and journal, approximating whatever he has found of value or that has withstood the test of clinical application. French divides his book into three general parts, the first embracing the principles of medicine; the second, dealing with practical medicine; and the third, describing clinical methods of examination.

Under the general division of principles of medicine the author considers the classification and causes of disease, pathology, and the bacteria of disease. The division relating to practical medicine is subdivided into ten sections, all dealing comprehensively with the general and clinical history of disease. In the final division in which clinical methods of examination are considered, the author writes most intelligently regarding examination of the blood, stomach-contents, intestinal discharges, urine, and sputum. This is a treatise that grows in favor with one as he examines it more and more, and we have somewhat reluctantly reached the conclusion that it is one of the most practical works extant dealing with the problems of internal medicine.

---

A Manual of the Practice of Medicine. By A. A. Stevens, A. M., M. D., Professor of Pathology in the Woman's Medical College of Pennsylvania. Sixth Edition, thoroughly revised, enlarged and reset. Post-octavo, 556 pages, illustrated. Philadelphia, New York, London: W. B. Saunders & Company. 1903. (Flexible leather, $2.25 net.)

Practitioners and students of medicine have become familiar with this popular manual, which first appeared in 1892. Its arrangement is excellent, being such as to facilitate ready reference to its multifarious subjects. The size of the book and its general make-up are of most convenient and substantial form. This edition has been thoroughly revised, the entire section on diseases of the digestive system, as well as many other articles having been rewritten. The importance of an accurate statement regarding our present knowledge of disorders of digestion cannot be overestimated, hence, the pains taken in that direction by the author will be appreciated by every reader of his book. We commend the manual as one of the cleverest yet presented on the practice of medicine.

---

Lea's Medical Epitome Series of Physics and Inorganic Chemistry. A Manual for Students and Practitioners. By A. McGlannan, M. D., Associate Professor of Physiological Chemistry, College of Physicians and Surgeons, Baltimore, Md. Duodecimo, pp. 216. Illustrated with twenty engravings. Series edited by V. C. Pedersen, A. M., M. D. Instructor in Surgery at the New York Polyclinic Medical School and Hospital. Lea Brothers & Co., Philadelphia and New York. 1903. Price, $1.00.)

The essentials of physics and inorganic chemistry are well arranged in this small volume. These two important topics are treated in a manner that will appeal to the student preparing for review or examination. There is no attempt at originality by the author, nor has he included doubtful or controverted questions. The aim is to present certain facts that lie at the foundation of these subjects in a manner that will attract the eye and impress the memory, and we think that he has succeeded in doing so most happily.

